# Off-label intravenous thrombolysis for early recurrent brain embolism associated with aortic arch thrombus

**DOI:** 10.1186/s42466-021-00103-6

**Published:** 2021-01-16

**Authors:** Ioanna Koutroulou, Georgios Tsivgoulis, Vasileios Rafailidis, Elissavet Psoma, Konstantinos Kouskouras, Panagiotis Fotiadis, Nikolaos Grigoriadis, Theodoros Karapanayiotides

**Affiliations:** 1 Second Department of Neurology, Aristotle University of Thessaloniki, School of Medicine, Faculty of Health Sciences, AHEPA University Hospital, Thessaloniki, Greece; 2 Second Department of Neurology, National & Kapodistrian University of Athens, “Attikon” University Hospital, Athens, Greece; 3 Department of Neurology, University of Tennessee Health Science Center, Memphis, TN USA; 4 Department of Radiology, Aristotle University of Thessaloniki, School of Medicine, Faculty of Health Sciences, AHEPA University Hospital, Stilponos Kyriakidi 1, 54636 Thessaloniki, Greece

**Keywords:** Off-label thrombolysis, Aortic arch thrombus, Stroke, Brain embolism

## Abstract

Safety data of intravenous thrombolysis (IVT) in presence of aortic arch thrombus is scant. Furthermore, IVT is debatable in patients with prior recent stroke. We present a 51-year-old woman with recurrent major infarction 5 days after a minor left MCA territory stroke. She had a floating aortic arch thrombus and she was treated safely and effectively with off-label IVT. Patients with small infarct volumes and mild/no residual neurological deficits after an initial stroke might be considered for IVT in case of early recurrence. IVT may be reasonable in a context of acute severely disabling stroke associated with aortic arch thrombus.

Aortic arch atheromatosis proximal to the ostium of the left subclavian artery has been established as a risk factor for recurrent stroke [1]. Safety data of intravenous thrombolysis (IVT) in presence of aortic arch thrombus is extremely scarce [2]. We present a patient with recurrent cerebral ischemia associated with aortic arch thrombus, treated safely and effectively with IVT.

## Case report

**Fig. 1:**
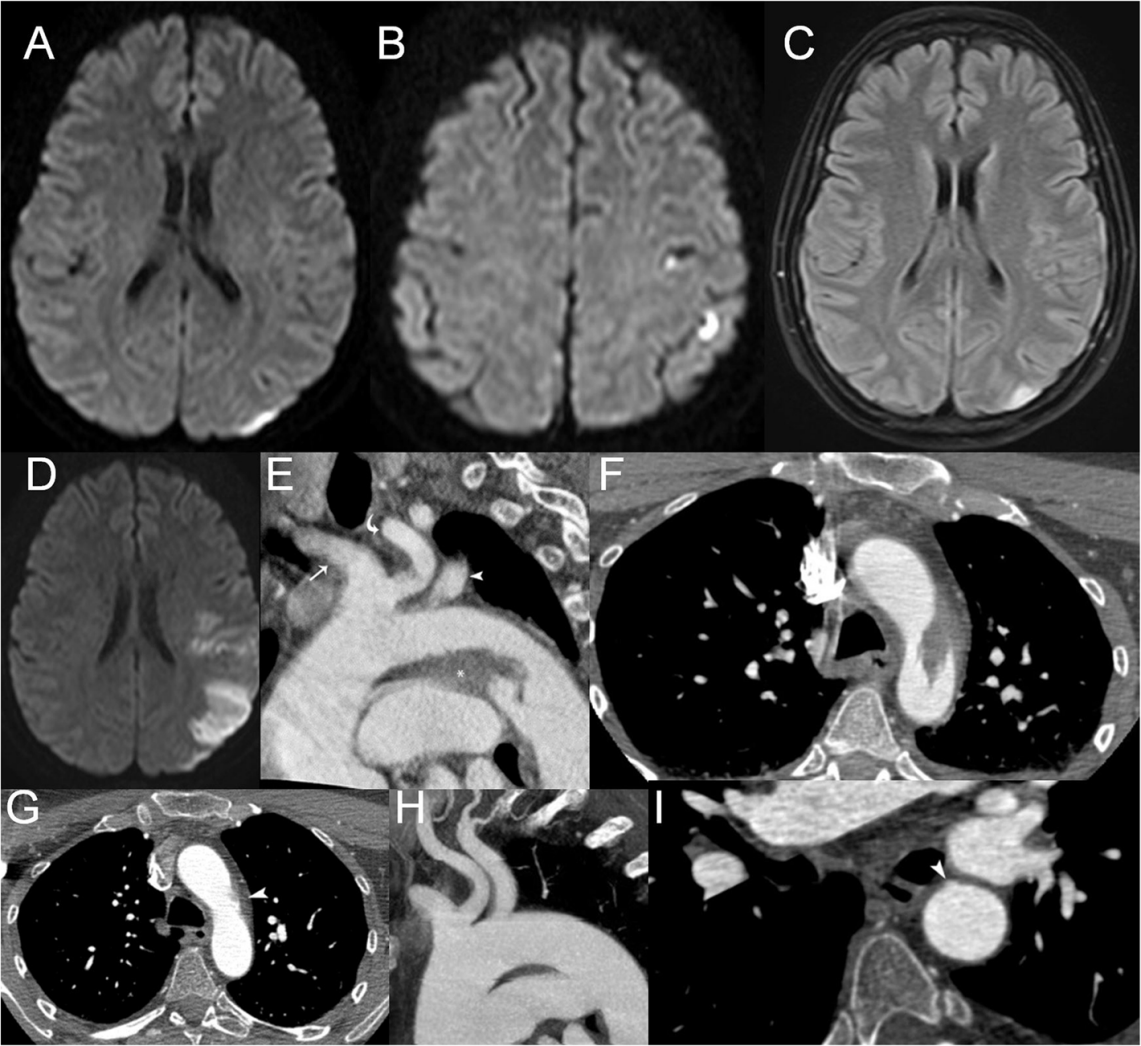
Baseline and follow-up brain MRI and CT angiography of the aortic arch. Figure Legend. Axial brain DWI showing minor foci of restricted diffusion in the parietal cortical territory of the left MCA (**a**-**b**)**.** There is total matching of the lesion size between DWI and FLAIR images (**c**). Repeat axial brain DWI showing a recurrent infarction in the posterior left MCA territory (**d**). Oblique sagittal reformatted CT angiography showing a broad-based thrombus (asterisk) situated at the inferior wall of the aortic arch, facing the origin of the great vessels. Notice the origin of the left common carotid artery (curved arrow) from the brachiocephalic trunk (arrow), so-called “bovine arch”, and the origin of the left subclavian artery (arrowhead) (**e**). Axial CT angiography showing the floating intraluminal thrombus as a flame-like filling defect attached to the wall of the aortic arch (**f**). Follow-up reformatted axial (**g**) and sagittal (**h**) CT angiography confirming complete dissolution of the thrombus after anticoagulation. Notice the mild subjacent atheroma (arrowhead) that nested the thrombus. Follow-up oblique axial CT angiography demonstrating an outpouching of the contrast medium (arrowhead), representing a shallow ulceration in the subjacent atheroma (**i**)

A 51-year-old woman was admitted with a transient episode of expressive aphasia. Apart from smoking her medical history was unremarkable. The neurologic examination was normal and the ABCD2 score was 2. Brain MRI revealed minor acute cortical infarctions in the left middle cerebral artery (MCA) territory (Fig. 1a-c) and no pathology of the intracranial and extracranial vessels. She was started on aspirin and 20 mg of atorvastatin. Transthoracic echocardiography and 48-h holter monitoring were unrevealing. Five days after admission she suffered from occlusion of the left MCA (NIHSS-score:23), confirmed by bedside transcranial Doppler. Brain CT showed only the known minor infarctions. Since mechanical thrombectomy (MT) was not available we proceeded to off-label IVT with alteplase, starting 80 min after the onset of symptoms and resulting within 24 h to near-complete reversal of the neurological deficit (NIHSS-score:1, moderate aphasia). Repeat MRI showed a recurrent infarction in the left posterior MCA territory (Fig. 1d). CT angiography of the aortic arch revealed a voluminous floating thrombus (Fig. 1e-f). She was discharged on acenocoumarol and CT angiography 40 days later documented complete dissolution of the thrombus and a small subjacent ulcerated atheroma (Fig. 1g-i). She was switched to aspirin and high-intensity statin therapy and 1 year later she suffers only from mild expressive aphasia.

## Discussion

Αortic arch disease may exist in more than one third of patients who do not have evidence of carotid disease [1]. In our patient, early stroke recurrence in the same vascular territory argued in favor of carotid disease missed during the initial assessment, or of some undiagnosed emboligenic pathology affecting the origin of the left carotid axis. Dual antiplatelet treatment early after minor stroke/TIA is superior to aspirin monotherapy [3] and together with high-dose statin might have averted thrombus generation on an unstable aortic arch atheroma; however, its potential effect on an already formatted voluminous thrombus was judged inferior to anticoagulation with acenocoumarol.

In view of the new emergent large vessel occlusion, MT represented the treatment of choice. However, even if MT was feasible at that time, there are no data on the safety of the procedure in presence of a mobile aortic arch thrombus. The German Neurological Society recommends that treatment decision should be made on a case-by-case basis, taking into account stroke etiology, infarct size, latency and severity [4]. A recent registry-based analysis found that only patients with stroke within the previous 2 weeks have higher rate of symptomatic intracerebral hemorrhage (sICH) or serious alteplase-related complications [5]. Conversely, the largest series of IVT despite recent prior stroke did not find different rates of sICH or functional independence [6]. A series of 19 patients who underwent IVT for both the index and early recurrent stroke had a median time interval of 30 days [7]. The study population comprised mainly of patients with excellent clinical recovery (median NIHHS: 1) and small infarct volumes (median: 1.5 cm^3^) in the MCA territory prior to the second thrombolysis. The repeated IVT was not complicated by sICH and functional independence was achieved in 47.4% of patients [7]. Moreover, a series of 25 patients treated with IVT for stroke occurring during hospitalization for TIA, reported no sICH and high recanalization rates [8]. Notably, the median interval from hospital admission to symptom recurrence was 24 h in this cohort. Additionally, recent silent infarctions did not increase the bleeding risk after IVT in a series of 113 patients [9].

Another issue pertains to the risk of a new embolic event, associated with intravenous fibrinolysis in presence of a large floating aortic arch thrombus, prone to fragmentation. Since relevant safety data are available for only three cases [2], we could extrapolate conclusions from patients with left-sided cardiac thrombi who underwent IVT; however, the relevant data is also limited and contradictory [10].

Although experience from a single case cannot be generalized, it would be logical to infer that IVT: a) may be considered in patients with small infarct volumes and mild/no residual deficits in case of early recurrence and b) may be reasonable for severely disabling stroke in a context of aortic arch thrombus.

## Data Availability

Not applicable.

## Abbreviations

IVT: Intravenous thrombolysis
MCA: Middle cerebral artery
MT: Mechanical thrombectomy
sICH: Symptomatic intracerebral hemorrhage
